# BoGHV-4 Genotypic Diversity Shapes Inflammatory and Viral Gene Expression in Platelet-Rich Plasma-Supplemented Bovine Endometrial Cells

**DOI:** 10.3390/v18010064

**Published:** 2025-12-31

**Authors:** Sofia López, Ignacio Álvarez, Santiago Delgado, Valentina Andreoli, Naiara Urrutia Luna, Marisol Yavorsky, Susana Pereyra, Stefano Grolli, Erika González Altamiranda, Sandra Pérez, Andrea Verna

**Affiliations:** 1 Instituto de Innovación para la Producción Agropecuaria y el Desarrollo Sostenible (IPADS) (INTA–CONICET), Ruta 226, km 73.5, Balcarce B7620, Buenos Aires, Argentinayavorsky.marisol@inta.gob.ar (M.Y.);; 2 Facultad de Ciencias Agrarias, Universidad Nacional de Mar del Plata, Ruta 226, km 73.5, Balcarce B7620, Buenos Aires, Argentina; sdelgado@usp.br; 3 Laboratorio Álvarez, 25 de Mayo 139, Bahía Blanca B8000, Buenos Aires, Argentina; 4 Escola Superior de Agricultura Luiz de Queiroz, Universidad de São Pablo, Piracicaba CEP 13418-900, Brazil; 5 Dipartimento di Scienze Medico Veterinarie, Università di Parma, Via del Taglio 8, 43126 Parma, Italy; valentina.andreoli@unipr.it (V.A.); stefano.grolli@unipr.it (S.G.); 6 Facultad de Ciencias Veterinarias, Universidad Nacional del Centro de la Provincia de Buenos Aires (CIVETAN), Tandil B7000GHG, Buenos Aires, Argentina

**Keywords:** *Bovine gammaherpesvirus 4*, platelet-rich plasma, cytokine modulation, lipopolysaccharide

## Abstract

*Bovine gammaherpesvirus 4* (BoGHV-4) is an opportunistic uterine pathogen whose reactivation is associated with postpartum inflammation and bacterial lipopolysaccharide (LPS). Platelet-rich plasma (PRP) is a regenerative biotherapeutic capable of modulating inflammatory responses, although its effects may vary depending on BoGHV4 genotype. In this study, primary bovine endometrial cells (BECs) were cultured in medium containing 10% PRP instead of fetal bovine serum, infected with two genetically divergent BoGHV-4 isolates (07-435, genotype 3; 10-154, genotype 2), and subsequently stimulated with bacterial lipopolysaccharide (LPS, 100 ng/mL). Expression of the viral immediate-early gene IE-2 and host immune genes (TLR4, TNF-α, CXCL8, and IFN-γ) were quantified by RT-qPCR from 4 to 48 h after stimulation. Isolate 07-435 induced a sustained activation of IE-2 and gradual cytokine upregulation, while isolate 10-154 elicited an early but transient inflammatory response followed by gene downregulation. PRP did not modify the strain-specific patterns of viral and inflammatory gene expression but established a common inflammatory baseline, whereas the magnitude and temporal profile of the response continued to be dictated by the viral genotype. These findings indicate that BoGHV-4 genotypic diversity remained the main determinant of response intensity and duration, supporting PRP as a context-dependent rather than a universal antiviral modulator.

## 1. Introduction

*Bovine gammaherpesvirus 4* (BoGHV-4) is a Rhadinovirus widely detected in cattle and capable of infecting endometrial epithelial and stromal cells [1]. Although its precise etiological role in uterine disease remains under discussion, increasing evidence supports a pathogen–environment interaction model in which postpartum bacterial contamination and inflammation contribute to viral reactivation. In this context, lipopolysaccharide (LPS)–mediated Toll-like receptor 4 (TLR4) signaling has been shown to reactivate latent BoGHV-4, enhance inflammation and disturb the endometrial microenvironment [2]. The BoGHV-4 immediate-early gene IE-2 is essential for the initiation of lytic replication and for transactivating host chemokines such as CXCL8, reinforcing inflammatory signaling within the uterus [3]. Thus, even if BoGHV-4 is not a standalone cause of endometritis, it may act as a relevant co-factor during inflammatory conditions.

BoGHV-4 exhibits considerable genetic diversity. Whole-genome sequencing of two Argentinean isolates—07-435 (genotype 3) and 10-154 (genotype 2)—revealed marked divergence from the genotype-1 reference strain, including differences in envelope glycoproteins (e.g., gp180) and immunomodulatory loci (e.g., Bo17), which may influence viral entry, immune evasion and antigenicity [4]. This heterogeneity provides a biologically plausible basis for genotype-specific patterns of infection and immune activation, suggesting that therapeutic modulation may not yield uniform outcomes across viral genotypes.

Current treatments for bovine endometritis rely mainly on antibiotics, which do not address viral cofactors, do not enhance mucosal repair, and contribute to antimicrobial resistance concerns and fertility impairment. Platelet-rich plasma (PRP)—an autologous biological product concentrated in platelets, growth factors, chemokines and antimicrobial peptides—has emerged as a non-antibiotic option with immunomodulatory and regenerative properties [5,6]. Specifically, PRP contains multiple key growth factors —such as TGF-β, PDGF, IGF-I, EGF, VEGF, FGF, and HGF— that act synergistically to promote immune cell infiltration, angiogenesis, matrix formation, and re-epithelialization, thereby enhancing tissue regeneration. Additionally, the presence of anti-inflammatory molecules such as HGF gives PRP the ability to attenuate inflammation [5]. Studies in bovine reproductive tissues show that PRP regulates inflammatory mediators, improves tissue repair, and modulates endometrial response to infection [7,8,9,10,11]. Additional reports in other species indicate that intrauterine PRP attenuates inflammation by affecting canonical pathways such as TLR4/NF-κB signaling and oxidative balance [12,13,14]. Collectively, these observations position PRP as a promising therapeutic strategy for supporting uterine recovery and reducing reliance on antibiotics in cattle.

Despite these advances, it remains unknown whether the immunomodulatory activity of PRP during BoGHV-4 infection is influenced by the viral genotype. The two isolates used in this study—07-435 (genotype 3) and 10-154 (genotype 2)—differ substantially in genomic regions associated with transcriptional regulation and immune evasion [4]. Previous work demonstrated that PRP modifies inflammatory and IE-2 expression in endometrial cells infected with isolate 07-435 [3] but signaling no comparative analysis has assessed whether these effects are conserved across genotypes.

We hypothesized that BoGHV-4 genotypic diversity modulates both the magnitude and the kinetics of the innate immune response in bovine endometrial cells (BECs) supplemented with PRP, resulting in genotype-specific viral and inflammatory transcriptional profiles. To test this hypothesis, we compared PRP-mediated regulation of the viral immediate-early gene IE-2 and key host immune genes (TLR4, TNF-α, CXCL8, and IFN-γ) in endometrial cells infected with both isolates. This experimental design enables assessment of whether the immunomodulatory effect of PRP is genotype-dependent, a critical consideration for its rational implementation in bovine reproductive management.

## 2. Materials and Methods

### 2.1. Viruses and Viral Stocks

Two BoGHV-4 strains representing divergent genotypes were employed. Strain 07-435, assigned to Genotype 3, and strain 10-154, classified as Genotype 2, were originally isolated in Argentina from the reproductive tract of cows with a history of abortion [4]. Both isolates were propagated in Madin–Darby bovine kidney (MDBK) cells minimum essential medium with Earle’s salts (MEM-E; Gibco, Thermo Fisher Scientific, Carlsbad, CA, USA) supplemented with 10% heat-inactivated foetal bovine serum (FBS; Bioser, Buenos Aires, Argentina), 1% penicillin–streptomycin, and 2 mM L-glutamine, under standard conditions of 37 °C and 5% CO_2_. Viral stocks were generated by infecting MDBK monolayers at a multiplicity of infection (MOI) of 0.5, and supernatants were collected prior to the onset of extensive cytopathic effect. To minimize contamination with host-derived material, supernatants were clarified by low-speed centrifugation (1000× *g*, 10 min, 4 °C) and further concentrated by ultracentrifugation (100,000× *g*, 2 h, 4 °C) through a 30% (*w*/*v*) sucrose cushion. The resulting pellets, containing enriched virions, were resuspended in serum-free MEM and stored at −80 °C until use. Viral infectivity was quantified in MDBK cells using endpoint dilution assays, and titres were expressed as the 50% tissue culture infectious dose per millilitre (TCID_50_/mL), calculated according to the Reed–Muench method [15].

### 2.2. Primary Culture of Bovine Endometrial Cells (BECs)

Bovine endometrial cells (BECs) were obtained from uteri collected at a local abattoir immediately after slaughter. Only reproductive tracts without macroscopic or clinical evidence of genital disease were selected to ensure sample quality, following previously described protocols [16]. Tissues were transported to the laboratory on ice and processed within 2 h of collection. Briefly, endometrial tissue fragments were dissected under sterile conditions, washed extensively with phosphate-buffered saline (PBS) containing antibiotics, and mechanically dissociated. The resulting cell suspensions were cultured in MEM-E supplemented with 10% FBS, 1% penicillin–streptomycin, and 2 mM L-glutamine. Cultures were maintained in a humidified incubator at 37 °C with 5% CO_2_ until reaching confluence.

To exclude contamination with common bovine reproductive pathogens, all cultures and FBS batches were screened for bovine alphaherpesvirus 1 (BoHV-1), bovine gammaherpesvirus 4 (BoGHV-4), and bovine viral diarrhea virus (BVDV), antigens by direct immunofluorescence (DIF) using FITC-labeled poly-clonal antibodies (IBR/BHV-1, CJ-F-IBR-mL; BVDV, CJ-F-BVD-mL, VMRD, Pullman, WA, USA), complemented by nested PCR or RT-PCR assays for nucleic acid detection, and confirmed by attempted viral isolation. Only pathogen-free BEC cultures were used for subsequent experiments.

### 2.3. Preparation of Platelet-Rich Plasma (PRP)

Was prepared from whole blood obtained from clinically healthy donor cows belonging to the INTA Balcarce herd, in accordance with institutional ethical guidelines (CICUAE protocol 232/2021). To reduce inter-individual variability, all PRP used in this study was derived from a single donor animal, previously screened and confirmed free of reproductive pathogens, including BVDV, BoHV-1, BoHV-5, BoGHV-4, *Brucella* spp., and *Escherichia coli*. PRP was incorporated into the culture medium at a final concentration of 10%, serving as the sole supplement in place of fetal bovine serum (FBS) for all experimental conditions and throughout the complete duration of the assays.

Blood was collected aseptically using sterile 60 mL syringes containing 5 mL of 4% sodium citrate as an anticoagulant. A maximum of 120 mL blood was drawn per donor. PRP was processed using a semi-automated closed system (Ematik^®^ SemiManual Kit, Prometheus Srl, Parma, Italy), strictly following the manufacturer’s instructions and previously published protocols [4,17]. The procedure involved two sequential centrifugation steps, after which platelets were concentrated and resuspended in a reduced volume of platelet-poor plasma (PPP). The final preparation was collected into sterile 1.5 mL tubes and stored at −20 °C until use. Quality control was performed by manual platelet counting in an aliquot of each preparation, which confirmed concentrations consistently within the range of 0.8 × 10^9^ to 1.3 × 10^9^ platelets/mL, ensuring standardization across experiments and reproducibility of biological activity.

### 2.4. Viral Infection

For infection assays, BECs were seeded at a density of 8 × 10^5^ cells per well in 6-well culture plates (Greiner Bio-One, Numbrecht, Germany) and maintained at 37 °C in a humidified atmosphere with 5% CO_2_. The culture medium was supplemented exclusively with 10% platelet-rich plasma (PRP) together with antibiotics (penicillin 100 IU/mL, streptomycin 100 µg/mL) and antifungal agent (amphotericin B, 25 µg/mL; Gibco, Grand Island, NY, USA). After 24 h, once monolayers reached confluence, cells were exposed to BoGHV-4 at a multiplicity of infection (MOI) of 0.5. Two divergent isolates were used in parallel: strain 07-435 (Genotype 3) and strain 10-154 (Genotype 2). Viral adsorption was allowed for 1 h, after which inocula were removed and replaced with fresh PRP-supplemented medium. To reproduce uterine inflammatory conditions, lipopolysaccharide (LPS; Escherichia coli O55:B5, Santa Cruz Biotechnology, Dallas, TX, USA) was incorporated into the culture medium 24 h after viral infection at a final concentration of 100 ng/mL. LPS remained in the cultures for the duration of the experiment. Cells and supernatants were collected at 4, 12, 24, and 48 h post-LPS addition.

**Figure 1:**
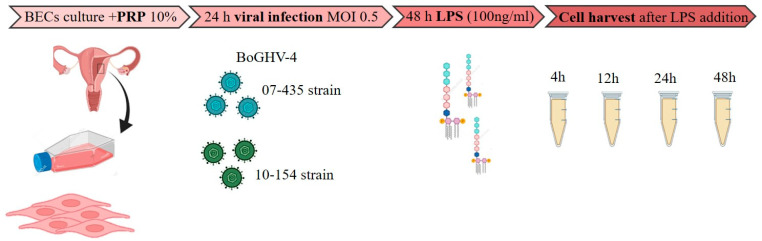
Schematic overview of experimental design. Primary bovine endometrial cells (BECs) were cultured in medium supplemented with 10% platelet-rich plasma (PRP). After 24 h, confluent monolayers were infected with BoGHV-4 isolates 07-435 (Genotype 3) or 10-154 (Genotype 2) at a multiplicity of infection (MOI) of 0.5. At 48 h post-infection, lipopolysaccharide (LPS, *E. coli* O55:B5, 100 ng/mL) was added to mimic bacterial co-factors associated with postpartum uterine infections. Cells were harvested at 4, 12, 24, and 48 h after LPS exposure for gene expression analysis by RT-qPCR.

Each experimental set included the following conditions: (i) uninfected BECs cultured in medium supplemented with PRP, (ii) uninfected BECs incubated with LPS, (iii) BECs infected with either viral strain in the absence of LPS (V), and (iv) infected BECs exposed to LPS (V+LPS). All assays were performed in triplicate to ensure reproducibility (Figure 1)

### 2.5. RT-qPCR Analysis

To evaluate the transcriptional response of BECs to BoGHV-4 infection under platelet-rich plasma (PRP) modulation, we quantified the relative expression of both viral and host genes. The selected panel included the immediate-early viral gene IE-2, which is essential for initiating lytic replication, and key host immune mediators involved in innate inflammatory signaling (TLR4, TNF-α, CXCL8, IFN-γ). Gene expression data were normalized using glyceraldehyde-3-phosphate dehydrogenase (GAPDH), a classical endogenous housekeeping gene widely employed in BECs studies due to its stable expression across treatments and experimental conditions [3,18].

BECs infected with BoGHV-4 strain 07-435 (Genotype 3) or 10-154 (Genotype 2), with or without subsequent LPS exposure, were harvested at defined timepoints and preserved in BIO-ZOL reagent (PB-L, Buenos Aires, Argentina) at −80 °C until processing. Total, RNA was extracted following the manufacturer’s instructions. RNA yield and integrity were assessed spectrophotometrically by absorbance ratios (A260/280 and A260/230) to ensure purity prior to downstream applications.

First-strand cDNA synthesis was performed using 0.5 µg of total RNA and the iScript™ cDNA Synthesis Kit (Bio-Rad Laboratories, Hercules, CA, USA), following the supplier’s protocol. The resulting cDNA was stored at −80 °C until further analysis.

Quantitative PCR was conducted on a CFX96 Touch Real-Time PCR Detection System (Bio-Rad) using SsoAdvanced Universal SYBR Green Supermix (Bio-Rad). Thermal cycling conditions were as follows: initial denaturation at 95 °C for 10 min, followed by 40 amplification cycles of 95 °C for 15 s and 60 °C for 1 min. Specificity of amplification was verified by melting curve analysis at the end of each run. Primer sequences for all target and reference genes are detailed in Table 1.

All reactions were performed in technical triplicates. Results were expressed as threshold cycle (Ct) values, and relative expression was calculated using the 2^−ΔΔCt^ method [18], normalized against GAPDH. Comparative analyses were performed across strains and treatment groups to capture potential genotype-dependent effects of PRP on viral and host transcriptional dynamics.

### 2.6. Experimental Design and Statistical Analysis

To investigate whether the modulatory effect of platelet-rich plasma (PRP) on BoGHV-4 infection is genotype-dependent, bovine endometrial cells (BECs) were infected with either strain 07-435 (Genotype 3) or strain 10-154 (Genotype 2) at a multiplicity of infection (MOI) of 0.5. Following viral adsorption and subsequent stimulation with lipopolysaccharide (LPS, *E. coli* O55:B5, 100 ng/mL), cells were collected at four post-stimulation time points (4, 12, 24, and 48 h). PRP (10%) was included as a constant supplement of the culture medium for all experimental groups and was therefore not considered a treatment factor.

The experimental design followed a factorial structure in which viral strain (07-435 vs. 10-154), treatment (cells mock, LPS, V, and V+LPS), and time (4, 12, 24, and 48 h) determined the transcriptional outcomes. Because each viral strain was evaluated in an independent BEC preparation, all statistical analyses were performed within each strain, using the corresponding unstimulated PRP-supplemented culture as the reference condition. This analytical framework enabled assessment of both temporal dynamics and treatment-specific transcriptional changes for each target gene.

All RT-qPCR reactions were performed in technical triplicates, and a time-matched uninfected, unstimulated control culture was systematically included in all experimental conditions to enable accurate normalization and comparison across treatments. Relative expression values were calculated using the 2^−ΔΔCt^ method [18]. Briefly, for each sample, ΔCt was obtained as Ct (target gene) − Ct (GAPDH). ΔΔCt was then computed as ΔCt (sample) − ΔCt (control), where the control corresponded to the uninfected, unstimulated BECs maintained in PRP-supplemented medium (mock/PRP-only) harvested at the same post-stimulation time point. Fold changes are therefore reported relative to this time-matched mock/PRP-only condition (2^−ΔΔCt^).

Statistical analyses were conducted in R software (v4.4.0, R Core Team, 2024 [19]). For each strain, a two-way ANOVA model with treatment and time as fixed factors was fitted to evaluate main effects and the treatment × time interaction. Because the interaction term was significant across genes, planned pairwise comparisons were conducted at each time point using Fisher’s protected least significant difference (LSD) test, with Bonferroni adjustment for multiple testing.

This approach enabled us to determine whether PRP, used as a uniform culture supplement, allows detection of conserved or genotype-specific modulatory effects on viral and host immune gene expression.

## 3. Results

### 3.1. TLR4 Expression

Toll-like receptor 4 (TLR4) is the principal pattern-recognition receptor mediating cellular responses to bacterial lipopolysaccharide and represents a central signaling hub at the interface between bacterial inflammation and herpesvirus reactivation in the bovine endometrium.

**Figure 2:**
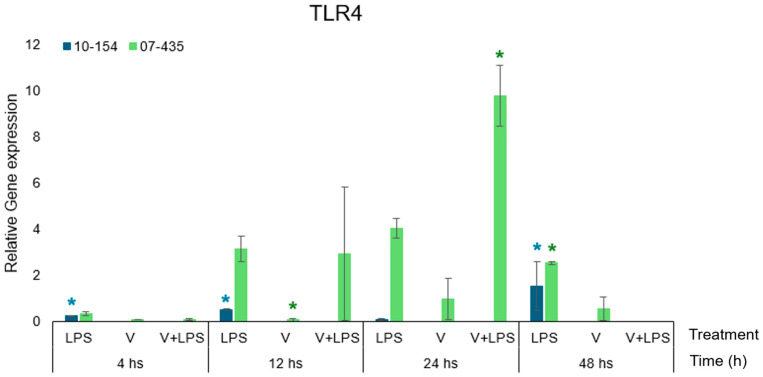
Relative expression of TLR4 in bovine endometrial cells cultured in PRP-supplemented medium and infected with BoGHV-4 strain 07-435 (Genotype 3, shown in green) or strain 10-154 (Genotype 2, shown in blue), with or without LPS co-stimulation. Data are expressed as mean ± SEM (fold change) across 4–48 h post-stimulation. For visual comparability, transcriptional responses for both viral strains are displayed side by side within the same figure; however, all statistical analyses were performed independently within each strain using the corresponding time-matched PRP-only condition as the control. Statistical significance (*p* < 0.05) was assessed by two-way ANOVA with treatment and time as fixed factors, followed by Fisher’s protected least significant difference (LSD) test with Bonferroni adjustment. The asterisks (*) in colours (green-strain 07-435, blue-strain 10-154) indicate statistically significant differences (*p* < 0.05) as a function of treatment and time.

TLR4 transcription showed marked genotype-dependent differences between the two BoGHV-4 isolates (Figure 2). In cells infected with strain 07-435 (genotype 3), TLR4 expression increased progressively over time, reaching its highest levels at 24–48 h under V+LPS stimulation. Virus alone induced moderate expression, whereas the addition of LPS resulted in strong enhancement.

In contrast, strain 10-154 (genotype 2) showed only modest changes in TLR4, with limited or no further induction upon LPS stimulation. Direct comparison demonstrated that strain 07-435 induced significantly higher TLR4 transcription than strain 10-154 at nearly all time points, particularly under V+LPS conditions.

PRP, used as the exclusive supplement in the culture medium across all experimental conditions, did not modify basal TLR4 expression nor alter the genotype-specific transcriptional profiles observed in infected cultures PRP, used as the exclusive supplement across all experimental conditions, did not alter basal TLR4 transcription when expression levels were analysed relative to the corresponding time-matched PRP-only control. Within this PRP-supplemented context, genotype-specific differences in TLR4 expression remained evident, indicating that transcriptional kinetics were dictated by the interaction between viral genotype and LPS rather than by PRP itself.

Even in the presence of PRP, strain-specific alterations in TLR4 expression remained evident, identifying viral genotype as the primary determinant of transcriptional activation. While PRP established a shared inflammatory baseline, the magnitude and temporal kinetics of TLR4 transcription were governed by the interaction between viral genotype and LPS [7,18,20].

### 3.2. TNF-α Expression

Tumor necrosis factor alpha (TNF-α) is a key pro-inflammatory cytokine involved in the initiation and amplification of innate immune responses in the bovine endometrium and has been implicated in herpesvirus-associated inflammatory pathology under bacterial co-stimulation.

**Figure 3:**
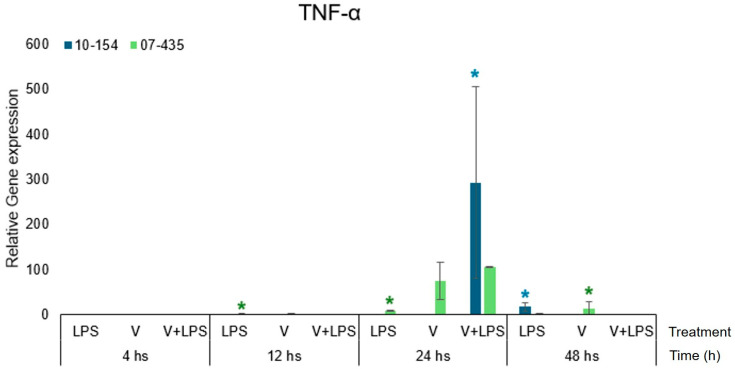
Kinetics of TNF-α expression in bovine endometrial cells cultured under PRP-supplemented conditions and infected with BoGHV-4 isolates (Genotype 3, shown in green) and strain 10-154 (Genotype 2, shown in blue). Data are expressed as mean ± SEM (fold change) across 4–48 h post-stimulation. For visual comparability, transcriptional responses for both viral strains are displayed side by side within the same figure; however, all statistical analyses were performed independently within each strain using the corresponding time-matched PRP-only condition as the control. Statistical significance (*p* < 0.05) was assessed by two-way ANOVA with treatment and time as fixed factors, followed by Fisher’s protected least significant difference (LSD) test with Bonferroni adjustment. The asterisks (*) in colours (green-strain 07-435, blue-strain 10-154) indicate statistically significant differences between treatments within the same strain at the corresponding time point.

Strain 07-435 elicited a robust and time-dependent induction of TNF-α (Figure 3). Transcript abundance increased at 12 h and continued to rise, reaching its highest levels at 24 h under V+LPS stimulation, where it remained elevated at 48 h.

In contrast, strain 10-154 displayed a markedly attenuated transcriptional profile (Figure 3), characterized only by a limited and transient increase under V+LPS at 24 h.

Across all experimental time points and conditions, strain 07-435 consistently induced higher TNF-α transcription than strain 10-154, demonstrating that the magnitude of inflammatory activation is strongly dependent on viral genotypes.

When expression levels were normalized to the time-matched PRP-only control, TNF-α transcription under V+LPS stimulation was reduced in PRP-supplemented cultures infected with strain 07-435. This attenuation reflects a lower magnitude of TNF-α induction within PRP-supported conditions, without altering the temporal profile or the genotype-dependent differences observed between viral isolates. Even in the presence of PRP, strain-specific differences in TNF-α transcription were preserved. Cultures infected with strain 07-435 displayed a higher and more sustained TNF-α response than those infected with strain 10-154, particularly under V+LPS stimulation. PRP reduced the overall magnitude of TNF-α induction but did not alter the temporal dynamics or the genotype-dependent divergence between isolates.

These results are consistent with the recognized role of TNF-α as a central mediator of endometrial inflammation and with previous reports indicating synergistic enhancement of TNF-α expression during concurrent herpesvirus infection and LPS exposure [20,21,22].

### 3.3. IE-2 Expression in PRP-Treated Bovine Endometrial Cells

The immediate-early gene IE-2 represents the earliest transcriptional checkpoint of BoGHV-4 reactivation and plays a central role in initiating lytic infection as well as in modulating host inflammatory gene expression.

**Figure 4:**
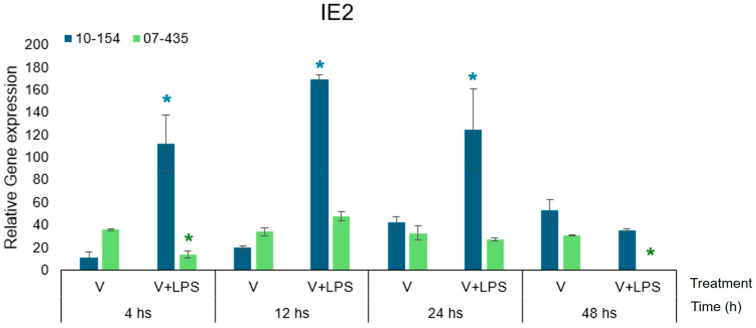
Temporal dynamics of the immediate-early viral gene IE2 in bovine endometrial cells cultured under PRP-supplemented conditions and infected with BoGHV-4 isolates (Genotype 3, shown in green) and strain 10-154 (Genotype 2, shown in blue). Data are expressed as mean ± SEM (fold change) across 4–48 h post-stimulation. For visual comparability, transcriptional responses for both viral strains are displayed side by side within the same figure; however, all statistical analyses were performed independently within each strain using the corresponding time-matched PRP-only condition as the control. Statistical significance (*p* < 0.05) was assessed by two-way ANOVA with treatment and time as fixed factors, followed by Fisher’s protected least significant difference (LSD) test with Bonferroni adjustment. The asterisks (*) in colours (green-strain 07-435, blue-strain 10-154) indicate statistically significant differences (*p* < 0.05) as a function of treatment and time.

IE-2 transcriptional dynamics differed markedly between the two viral isolates (Figure 4).

Strain 07-435 (genotype 3) exhibited an early and sustained upregulation of IE-2, evidenced by increased transcript abundance as early as 4 h, which remained elevated through 48 h. This profile consists of a continuous progression of lytic transcription.

In contrast, strain 10-154 (genotype 2) displayed a transient yet markedly amplified increase in IE-2 expression, particularly under V+LPS stimulation between 12–24 h, followed by a pronounced decline in transcript levels at 48 h.

Even in the presence of PRP, the transcriptional behavior of the immediate-early gene IE-2 differed markedly between viral strains. PRP did not modify the sustained activation profile of strain 07-435 nor the transient expression pattern observed in strain 10-154, indicating that IE-2 regulation is primarily dictated by viral genotype rather than by PRP supplementation (Figure 4).

Overall, these results reveal that BoGHV-4 genotype shapes the IE-2 transcriptional phenotype, yielding either a persistent activation profile (07-435) or a transient response potentiated by LPS (10-154).

### 3.4. CXCL8 Expression

CXCL8 is a major chemokine involved in neutrophil recruitment and inflammatory cell trafficking in the postpartum uterus and constitutes a well-established downstream target of TLR4- and IE-2–dependent signaling pathways.

**Figure 5:**
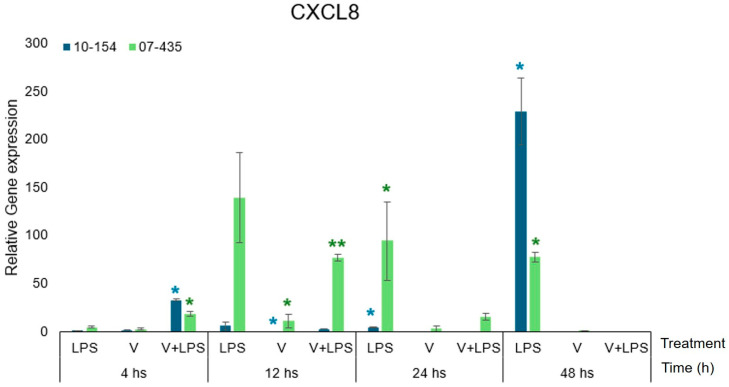
Comparative transcriptional kinetics of CXCL8 in bovine endometrial cells cultured in PRP-supplemented medium and infected with BoGHV-4 strain 07-435 (Genotype 3, shown in green) or strain 10-154 (Genotype 2, shown in blue), with or without LPS co-stimulation. Data are expressed as mean ± SEM (fold change) across 4–48 h post-stimulation. For visual comparability, transcriptional responses for both viral strains are displayed side by side within the same figure; however, all statistical analyses were performed independently within each strain using the corresponding time-matched PRP-only condition as the control. Statistical significance (*p* < 0.05) was assessed by two-way ANOVA with treatment and time as fixed factors, followed by Fisher’s protected least significant difference (LSD) test with Bonferroni adjustment. The asterisks (* and **) in colours (green-strain 07-435, blue-strain 10-154) indicate statistically significant differences (*p* < 0.05) as a function of treatment and time.

CXCL8 expression mirrored the genotype-dependent patterns observed for TLR4 and TNF-α (Figure 5).

Strain 07-435 elicited a robust CXCL8 response, reaching its highest transcript abundance between 12–24 h under V+LPS stimulation, whereas strain 10-154 exhibited low or variable transcription, with appreciable induction only under V+LPS.

Within PRP-supplemented conditions and relative to the time-matched PRP-only baseline, CXCL8 overexpression was reduced in 07-435–infected cultures under V+LPS stimulation. Importantly, PRP supplementation did not alter the timing of CXCL8 induction nor the pronounced genotype-dependent differences between viral isolates.

Even in the presence of PRP, CXCL8 transcription mirrored the strain-specific inflammatory profiles observed for TLR4 and TNF-α. Although PRP attenuated the overall magnitude of CXCL8 induction, the timing and genotype-dependent differences between viral isolates remained unchanged [23,24].

### 3.5. IFN-γ Expression

Interferon gamma (IFN-γ) is a key immunoregulatory cytokine involved in antiviral defense and inflammatory homeostasis in the bovine endometrium, and its expression reflects the balance between viral activation and host immune control.

**Figure 6:**
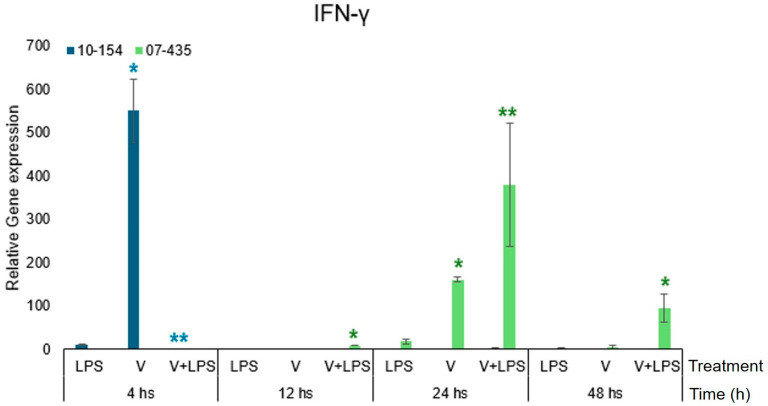
Time-dependent IFN-γ expression bovine endometrial cells cultured in PRP-supplemented medium and infected with BoGHV-4 strain 07-435 (Genotype 3, shown in green) or strain 10-154 (Genotype 2, shown in blue), with or without LPS co-stimulation. Data are expressed as mean ± SEM (fold change) across 4–48 h post-stimulation. For visual comparability, transcriptional responses for both viral strains are displayed side by side within the same figure; however, all statistical analyses were performed independently within each strain using the corresponding time-matched PRP-only condition as the control. Statistical significance (*p* < 0.05) was assessed by two-way ANOVA with treatment and time as fixed factors, followed by Fisher’s protected least significant difference (LSD) test with Bonferroni adjustment. The asterisks (* and **) in colours (green-strain 07-435, blue-strain 10-154) indicate statistically significant differences (*p* < 0.05) as a function of treatment and time.

IFN-γ transcription differed markedly between viral isolates (Figure 6).

Strain 07-435 induced a delayed yet sustained transcriptional upregulation, which was further amplified by LPS and persisted up to 48 h. In contrast, strain 10-154 triggered an early and pronounced increase in IFN-γ transcript abundance at 4 h, followed by a rapid return to basal transcriptional levels.

When analysed relative to the corresponding PRP-only control, IFN-γ transcript levels showed a moderate reduction in magnitude under PRP-supplemented conditions for both viral strains. However, PRP did not affect the distinct temporal dynamics imposed by each genotype, which remained the principal determinant of IFN-γ transcriptional behaviour.

Even in the presence of PRP, IFN-γ transcription followed distinct genotype-dependent trajectories. PRP moderated the magnitude of IFN-γ expression in both strains but did not alter the delayed and sustained profile induced by strain 07-435 or the early and transient response characteristic of strain 10-154 [24,25].

## 4. Discussion

BoGHV-4 infection in the postpartum endometrium is increasingly understood as a multifactorial process in which viral activity depends on the inflammatory context generated by bacterial contamination. An important aspect to consider when interpreting the present results is the transcriptional behaviour observed in LPS-only–treated bovine endometrial cell cultures. In this study, lipopolysaccharide was not implemented as a static reference or negative control, but rather as an active inflammatory stimulus intended to reproduce the bacterial component of postpartum uterine inflammation. In primary bovine endometrial cells, LPS triggers TLR4-dependent signaling pathways whose magnitude and temporal dynamics are inherently time-dependent and context-sensitive, even in the absence of viral infection. Consequently, variation in LPS-induced gene expression across time points reflects the expected biological dynamics of innate immune activation rather than uncontrolled experimental variability. From a methodological standpoint, transcriptional responses were analyzed relative to a time-matched uninfected and unstimulated control (mock/PRP-only), which served as the control condition for 2^−ΔΔCt^ calculation. This analytical approach enables each experimental condition, including (LPS-only) to be interpreted in relation to its corresponding baseline at each time point, rather than assuming absolute stability of LPS-induced responses across experimental sets. Importantly, LPS-only conditions were not used to establish direct inter-strain comparisons; instead, analyses were conducted within each strain-specific experimental context to determine whether viral infection, alone or in combination with LPS, altered the magnitude or temporal profile of host and viral gene expression. Biologically, the dynamic nature of LPS-driven transcription observed here is consistent with previous studies demonstrating that endotoxin exposure elicits transient or progressive inflammatory responses in bovine endometrial cells depending on exposure time, signaling feedback mechanisms, and cellular context. Within this experimental setting, the genotype-dependent differences reported in the present study arise from the presence and persistence of viral infection and its interaction with LPS-induced signaling, rather than from variability intrinsic to LPS stimulation itself. This interpretation reinforces the conclusion that BoGHV-4 genotype, rather than LPS alone, is the principal determinant of the divergent transcriptional patterns observed in PRP-supplemented endometrial cell cultures.

Within this biological and analytical context, the inflammatory microenvironment generated by bacterial endotoxin constitutes a critical determinant of BoGHV-4 activity in the bovine endometrium, in which LPS from Gram-negative bacteria activates TLR4, triggering downstream NF-κB/MAPK signaling and generating an environment that favours herpesvirus reactivation in endometrial epithelial and stromal cells. In vitro, studies using primary BECs showed that BoGHV-4 and LPS act synergistically on TLR4-dependent pathways, inducing transcription of inflammatory mediators such as CXCL8 and IFN-γ [26]. Complementarily, recent studies demonstrated that PRP can modulate inflammatory gene expression and viral replication dynamics in bovine reproductive tissues, indicating that platelet-derived factors influence virus–host interactions in a concentration- and context-dependent manner [3,7]. This evidence provided justification for examining whether PRP-induced modulation differs between BoGHV-4 genotypes.

The two BoGHV-4 isolates analyzed in this study exhibited markedly different transcriptional behaviours, indicating that viral genotype constitutes a major determinant of the inflammatory response in bovine endometrial cells. Interpretation of the divergent transcriptional profiles observed between strains 07-435 and 10-154 must be considered in light of previously reported strain-specific genomic and biological characteristics. Comparative genomic analyses have demonstrated that these isolates differ across multiple loci implicated in viral regulation, immune interaction, and replication dynamics, including envelope glycoproteins, the immediate-early regulatory region, and immunomodulatory genes such as Bo17. These genomic differences have been associated with distinct phenotypic properties, including differential neutralizing antibody induction, immune recognition, and infection kinetics across host species. Although the present study did not address gene-specific functional validation, the temporal patterns of IE-2 transcription reported here are biologically consistent with these established strain-dependent regulatory features. Sustained IE-2 activation in strain 07-435 and transient IE-2 expression in strain 10-154 are more plausibly explained by differences in viral regulatory architecture and host–virus interaction dynamics than by isolated effects attributable to individual genetic elements. Within this context, the present findings support a genotype-associated transcriptional phenotype while deliberately avoiding direct causal attribution to specific viral loci, thereby underscoring the need for future studies employing targeted genetic approaches to further delineate the contribution of individual determinants to BoGHV-4 transcriptional regulation.

Consistent with this interpretation, strain 07-435 (genotype 3) induced a sustained transcriptional pattern characterized by continuous activation of the immediate-early gene IE-2 and progressive increases in TLR4, TNF-α, CXCL8, and IFN-γ, particularly under LPS stimulation. These results align with previous evidence demonstrating that IE-2 functions as a transcriptional activator of lytic replication and chemokine expression in endometrial cells [3,18,26]. In contrast, strain 10-154 (genotype 2) exhibited a short-duration transcriptional profile, in which IE-2 expression was transient and largely dependent on LPS, CXCL8 expression was low or intermittent, TLR4 remained minimally induced, and IFN-γ rapidly returned to basal levels. These observations are consistent with reports indicating that endotoxin amplifies inflammatory transcription only within defined temporal windows in the endometrium [7,27,28]. Comparative genomic analyses of BoGHV-4 further support this interpretation, demonstrating that isolates differ in envelope glycoproteins and regulatory loci—particularly ORF50/IE-2—which influence viral entry, transcriptional activation, and immune modulation [4,29,30,31,32]. Collectively, these findings indicate that genotypic variability underlies the divergent transcriptional outcomes observed in bovine endometrial cells

The interaction between LPS and BoGHV-4 further clarified the biological basis underlying these differences. In strain 07-435, LPS enhanced cytokine transcription and maintained increased transcript abundance over time, a pattern compatible with continuous IE-2 activation lowering the signaling threshold required for NF-κB/MAPK-driven transcription [1,3,21]. In strain 10-154, however, LPS triggered only brief increases in IE-2 and CXCL8 and did not restore TLR4 expression or sustain IFN-γ transcription. These observations indicate that the effect of LPS–TLR4 stimulation depends on the viral genotype and on the duration of IE-2 activation [7,28,29,32]. Thus, isolates capable of maintaining IE-2 activation engage in prolonged inflammatory transcription, whereas isolates with transient IE-2 expression rapidly return to baseline.

PRP reduced the magnitude of inflammatory transcription but did not modify the transcriptional pattern imposed by each genotype. PRP did not induce TLR4 expression and did not transform a short-duration profile into a sustained one, or vice versa. These findings align with evidence showing that platelet-derived products modulate inflammatory signaling in reproductive tissues without uniformly suppressing virus- or LPS-induced transcription [3,13,16,33]. PRP contains bioactive mediators—including TGF-β, PDGF, VEGF, PF-4/CXCL4 and extracellular vesicles—that regulate cytokine expression and leukocyte recruitment in a manner dependent on the inflammatory environment [13,26,33]. Under the experimental conditions (PRP 10% replacing fetal bovine serum), the viral genotype and LPS–TLR4 signaling remained the dominant regulators of transcription, whereas PRP selectively modulated transcriptional intensity.

An additional methodological aspect raised by the reviewer concerns the absence of a fetal bovine serum (FBS)–supplemented control group. This choice was deliberate and aligned with the central objective of the study, which was not to compare PRP against conventional serum supplementation, but to determine whether the immunomodulatory effect exerted by PRP is influenced by BoGHV-4 genotypic diversity under standardized culture conditions. By replacing FBS with PRP across all experimental groups, we established a controlled and biologically relevant environment in which PRP constituted a constant contextual factor rather than an experimental variable. This design enabled direct comparison of genotype-dependent viral and host transcriptional responses without the confounding effects introduced by serum-derived growth factors that are unrelated to platelet biology. Consequently, differences observed between viral isolates cannot be attributed to differential baseline culture conditions but rather reflect intrinsic genotype-specific regulatory properties operating within a PRP-supported inflammatory context.

With respect to the relationship between inflammatory gene induction and viral replication capacity, it is important to clarify that the present study was not designed to quantify productive viral replication or to generate growth kinetic curves in bovine endometrial cells. Instead, our primary focus was the regulation of the immediate-early gene IE-2, which constitutes the earliest transcriptional checkpoint of BoGHV-4 reactivation and a key determinant of downstream inflammatory signaling. IE-2 expression precedes viral DNA replication and virion production and therefore provides mechanistic insight into genotype-specific regulatory behaviors independently of later replication outcomes. While differences in replication kinetics may indeed emerge at subsequent stages of infection, the sustained versus transient IE-2 expression profiles observed here indicate that genotype-dependent divergence is already established at the level of immediate-early transcription. In this context, the inflammatory phenotypes reported cannot be interpreted solely as a consequence of viral load but rather reflect fundamental differences in early regulatory dynamics governing host–virus interaction. Future studies incorporating viral growth curves in parallel with transcriptional profiling will be valuable to further integrate regulatory and replicative dimensions of BoGHV-4 infection, but such analyses extend beyond the scope of the present work.

From a methodological perspective, the use of a single PRP concentration represents an additional consideration when interpreting the present findings. In this study, PRP was applied at a fixed concentration (10%, replacing fetal bovine serum) across all experimental conditions to ensure internal consistency and to allow direct comparison of transcriptional responses between viral genotypes and inflammatory contexts. While this approach enabled robust assessment of genotype-dependent effects under PRP-supported conditions, it does not permit evaluation of potential dose-dependent modulation of inflammatory or viral gene expression. Consequently, the present design cannot determine whether the selected concentration represents an optimal biological range, nor can it address potential synergistic or antagonistic interactions between PRP concentration and viral genotype. These aspects warrant further investigation in future studies incorporating PRP concentration gradients to refine culture conditions and better define the functional window of PRP-mediated immunomodulation.

Within this experimental and interpretative context, PRP should not be interpreted as establishing a neutral or suppressive state, but rather as defining a shared inflammatory context in which genotype-specific transcriptional programs could be compared. The observation that PRP reduced the overall magnitude of inflammatory transcription without altering the temporal pattern imposed by each viral genotype suggests that platelet-derived mediators modulate inflammatory signaling thresholds rather than dictating transcriptional trajectories. Although the present study did not directly interrogate molecular intermediates such as post-translational regulation of inflammatory mediators, growth factor–dependent signaling pathways, or immune cell recruitment, the results are consistent with the concept that PRP exerts context-dependent immunomodulatory effects that attenuate inflammatory intensity while preserving virus-driven regulatory dynamics.

An additional limitation of this study relates to biological variability associated with the use of PRP derived from a single clinically healthy donor. Although this strategy was deliberately adopted to minimize experimental heterogeneity and enhance reproducibility, it may constrain extrapolation of the findings to PRP preparations obtained from animals with differing physiological backgrounds. Furthermore, intrinsic host-related factors, including breed, age, and reproductive or metabolic status, were not addressed in the present experimental design and may influence the responsiveness of bovine endometrial cells to both viral infection and PRP supplementation. Such variables have the potential to affect not only the magnitude but also the kinetics of inflammatory transcriptional responses, thereby limiting the universality of the conclusions. Future investigations incorporating PRP from multiple donors and endometrial cells derived from animals with diverse biological characteristics will be essential to strengthen the robustness and translational relevance of these observations.

In summary, BoGHV-4 genotypic diversity determines whether endometrial transcriptional responses adopt a sustained pattern (strain 07-435) or a short-duration pattern (strain 10-154) in the presence of PRP and LPS. IE-2 persistence is associated with prolonged transcription of inflammatory mediators, whereas transient IE-2 activity leads to rapid resolution of expression. PRP decreases the magnitude of transcriptional activation but does not alter the expression pattern imposed by the viral genotype. These observations indicate that PRP acts as an immunomodulator rather than a suppressor, and reinforces a multifactorial framework in which viral genetics, bacterial endotoxin signaling and platelet-derived mediators jointly regulate the balance between immune activation and tissue restoration [3,21,26,34].

## 5. Conclusions

This study demonstrates that genotypic variability in BoGHV-4 is the principal factor determining the transcriptional and inflammatory responses of BECs exposed to PRP and LPS. The two isolates analyzed exhibited clearly distinct transcriptional profiles. Strain 07-435 (genotype 3) induced a sustained response characterized by persistent activation of the immediate-early gene IE-2 and progressive upregulation of TLR4, TNF-α, CXCL8 and IFN-γ. Under identical conditions, strain 10-154 (genotype 2) produced a rapid and short-duration response, defined by a transient increase in IE-2 and TNF-α followed by an early decline in chemokine and interferon transcription.

PRP reduced the magnitude of inflammatory gene expression but did not modify the temporal pattern established by each viral genotype. These findings indicate that the viral genetic background governs transcriptional outcome, whereas LPS/TLR4 signaling amplifies cytokine expression and PRP modulates its intensity without altering the genotype-dependent expression pattern. By linking viral genetic diversity with differential cellular responses to PRP, this work provides experimental evidence supporting the incorporation of genotype information into therapeutic decision-making in bovine reproductive medicine.

These findings highlight that genotype-dependent divergence in BoGHV-4 infection emerges at the level of immediate-early transcription, prior to productive replication, reinforcing the relevance of early regulatory checkpoints as therapeutic targets.

**Figure 7:**
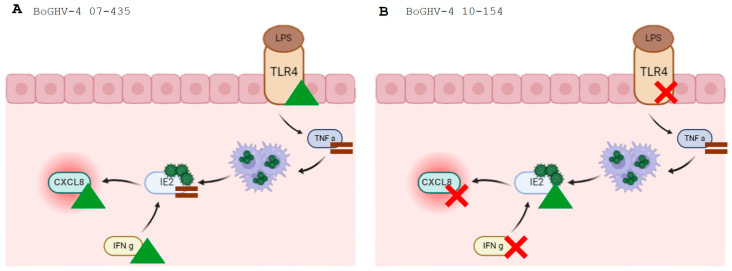
Differential modulation of inflammatory signaling in PRP-treated bovine endometrial cells infected with BoGHV-4 and stimulated with LPS. (**A**) Infection with BoGHV-4 strain 07-435 (Genotype 3) enhances TLR4 activation and promotes CXCL8 and IFN-γ expression in the presence of LPS. (**B**) Infection with BoGHV-4 strain 10-154 (Genotype 2) suppresses TLR4 signaling and downregulates CXCL8 and IFN-γ responses under the same conditions. Green triangles indicate stimulated expression, red crosses indicate inhibition expression, and brown equals signs indicate no significant change.

Importantly, the observation that PRP attenuates excessive inflammatory activation without suppressing essential immune responses highlights its potential as a supportive, non-antibiotic therapeutic strategy. Genotype-guided use of PRP may contribute to restoring uterine immune homeostasis in postpartum cows while reducing unnecessary antibiotic interventions and supporting antimicrobial stewardship in livestock systems, Figure 7.

## Figures and Tables

**Table 1:** Primer sequences used for RT-qPCR analysis of viral (IE-2) and host genes (TLR4, TNF-α, CXCL8, and IFN-γ) in bovine endometrial cells. GAPDH served as the endogenous control for normalization.

Gene	Primer Type	Sequence (5′–3′)
GAPDH	FWD	TTC TGC CAA AGT GGA CAT CGT
	REV	CCT GAC TGT GCC GTT GAA CTT
CXCL8	FWD	CCT CTT GTT CAA TAT GAC TTC CA
	REV	GGC CCA CTC TCA ATA ACT CTC
TNF-α	FWD	CCA CGT TGT AGC CGA CAT CA
	REV	CTG GTT GTC TTC CAG CTT CAC A
IE-2	FWD	ACA AAC ACA CAG ACC AGT CA
	REV	GTT TCA CAA CAG ATT GAG CA
TLR4	FWD	TGC CTT CAC TAC AGG GAC TTT
	REV	AGT GTC GCT GTT GAA GTC
IFN-γ	FWD	CAG CTC TGA GAA ACT GGA GGA CTT
	REV	GTG GCT GGA GTG GTT ATT AG

## Data Availability

Data are contained within the article.

## Abbreviations

The following abbreviations are used in this manuscript:

BoGHV-4	BovineGammaherpesvirus Type 4
PRP	Platelet-rich plasma
LPS	Lipopolysaccharide
TLR4	Toll-like receptor 4
IE-2	Viral immediate–early gene Viruses
TNF-α	Tumor necrosis factor-alpha
CXCL-8	Interleukin-8
IFN-γ	Interferon-gamma
BEC	Bovine endometrial cells
FBS	Fetal bovine serum
MOI	Multiplicity of infection
PBS	Phosphate-buffered saline
BoAHV-1	Bovine Alphaherpesvirus 1
BVDV	Bovine Viral Diarrhea virus
MDBK	Madin–Darby Bovine Kidney
Hpi	hours post-infection
PPP	Platelet-poor plasma
GAPDH	Glyceraldehyde-3-phosphate dehydrogenase
Ct	Threshold cycle
V+LPS	infected BECs exposed to LPS
